# Does Movement Matter? Prefrontal Cortex Activity During 2D vs. 3D Performance of the Tower of Hanoi Puzzle

**DOI:** 10.3389/fnhum.2019.00156

**Published:** 2019-05-10

**Authors:** Kimberly Milla, Elham Bakhshipour, Barry Bodt, Nancy Getchell

**Affiliations:** ^1^Biomechanics and Movement Sciences Interdisciplinary Program, University of Delaware, Newark, DE, United States; ^2^Developmental Motor Control Laboratory, Department of Kinesiology and Applied Physiology, University of Delaware, Newark, DE, United States; ^3^Biostatistics Core, College of Health Sciences, University of Delaware, Newark, DE, United States

**Keywords:** executive function, Tower of Hanoi, fNIRS, motor learning, motor planning

## Abstract

In the current study, we used functional near-infrared spectroscopy (fNIRS) to compare prefrontal cortex (PFC) activity in adults as they performed two conditions of the Tower of Hanoi (ToH) disk-transfer task that have equivalent executive function (EF) but different motor requirements. This study explored cognitive workload, here defined as the cognitive effort utilized while problem-solving by performance output. The first condition included a two-dimensional (2D) computerized ToH where participants completed trials using a computer mouse. In contrast, our second condition used a traditional, three-dimensional (3D) ToH that must be manually manipulated. Our aim was to better understand the role of the PFC in these two conditions to detect if PFC activity increases as a function of motor planning. Twenty right-handed, neurotypical adults (10M/10F, $\bar{x}$ = 24.6, SD ± 2.8 years old) participated in two blocks (one per condition) of three 1-min trials where they were asked to solve as many puzzles as possible. These data were analyzed using a mixed effects ANOVA with participants nested within blocks for 2D vs. 3D conditions, presentation order (leading block), individual participants, and regions and additional follow-up statistics. Results showed that changes in oxygenated hemoglobin, ΔHbO, were significantly higher for 3D compared to 2D condition (*p* = 0.0211). Presentation order and condition interacted significantly (*p* = 0.0015). Notably, a strong correlation between performance and ΔHbO existed between blocks 1 and 2 (*r* = −0.69, *r*^2^ = 0.473, *p* < 0.01) when the 3D condition was initially performed, in contrast to the 2D condition where no significant correlation was seen. Findings also showed a significant decrease in ΔHbO between the first and second block (*p* = 0.0015) while performance increased significantly for both 3D and 2D conditions (*p* < 0.005). We plan to use this information in the future to narrow the potential points of impairment on the perception-cognition-action continuum in certain developmental disabilities.

## Introduction

The prefrontal cortex (PFC) is a brain structure with a significant function in planning complex cognitive behavior, personality expression, decision making, and moderating social behavior (Yang and Raine, 2009). Processes underlying high-level executive function (EF) have historically been associated with the PFC (Ball et al., 2011). Furthermore researchers have identified neural networks that are active during complex executive processes and are highly interconnected between the PFC and several brain regions, such as the posterior parietal cortex, limbic, and cortical areas (Selemon and Goldman-Rakic, 1988; Öngür and Price, 2000; Halgren et al., 2002; Honey et al., 2002; Schall et al., 2003; Periáñez et al., 2004; Owen et al., 2005; Simmonds et al., 2008). EF is an umbrella term encompassing the higher-level cognitive processes responsible for the completion of a specific goal and its related behaviors such as anticipation, goal establishment, monitoring of results, inhibition of action, and planning (Stuss, 1986; Moriguchi and Hiraki, 2013).

The role of the PFC in motor planning is to outline the execution of programmed sequences of actions and to plan the consequences of such actions, also referred to as internal modeling (Kawato, 1999). Specifically, the PFC is concerned with the active representation of future events resulting from behavioral actions within a framework of problem-solving, with the dorsolateral PFC particularly suited to assist in the control of responses to environmental stimuli and regulation of behavior (Wood and Grafman, 2003; Mushiake et al., 2009).

The experimental evidence for a better understanding of EF is the result of using different tasks such as the Stroop Task, where the ink of a word representing a color might mismatch such color (Smith et al., 2006), the Wisconsin Card Sorting Task, where participants classify cards based on different criteria (Koch et al., 2018), and the Tower of Hanoi puzzle (ToH), a disk-transfer task that requires the relocation of disks from an initial configuration to a target configuration in the least possible number of moves (Liang et al., 2016b). ToH requires participants to appropriately respond to new situations, as well as demands related to anticipatory, means-end problem-solving (Welsh and Huizinga, 2001). Moreover, it has been used for decades as a neuropsychological task for assessing EF on healthy individuals and different clinical populations (Goel and Grafman, 1995; Slomine et al., 2002; Griebling et al., 2010; Yu et al., 2016; Shuai et al., 2017). Previous research utilizing Tower tests have all resulted in PFC activity, including the anterior, inferior, and dorsolateral regions, despite the use of different parameters (i.e., the amount, size, and color of the disks used; Baker et al., 1996; Boghi et al., 2006; Wagner et al., 2006; Just et al., 2007; den Braber et al., 2008; Fitzgerald et al., 2008; Campbell et al., 2009; Zhu et al., 2010; Kaller et al., 2011; Stokes et al., 2011; de Ruiter et al., 2009; Hahn et al., 2012; Ruocco et al., 2014). Furthermore, an increase in the difficulty of the task (i.e., increased number of moves necessary to reach the target state) led to increased levels of activity, particularly in the left dorsolateral region of the PFC (dlPFC). The left dlPFC has been related to the identification of information relevant to the goal and creation of an internal problem representation, whereas the right dlPFC has been associated with mental transformations and working memory (Ruocco et al., 2014).

Notably, the ToH puzzle has the advantage of being sensitive to disruption in the PFC (Simon, 1975; Saint-Cyr et al., 1988; Casey et al., 1994; Goel and Grafman, 1995). Additionally, the activity of the PFC during the computerized version of ToH has been shown using different neuroimaging techniques such as fMRI (Head et al., 2002; Griebling et al., 2010; Crescentini et al., 2012) and EEG (Ruiz-Díaz et al., 2012; Guevara et al., 2013), in addition to functional near-infrared spectroscopy (fNIRS; Liang et al., 2016a,b). fNIRS is a neuroimaging tool that is non-invasive, portable, affordable, and safe for continuous and repeated measurements. fNIRS indirectly measures neural activity using the neurovascular coupling principle (i.e., blood flow follows neural activity). This phenomenon occurs when increases in neural activity also increase the delivery of oxygen and glucose for consumption, leading to closely associated increases in blood flow. Ultimately, the increased blood flow into the capillary beds facilitates access to oxygenated hemoglobin in the local tissue. Thus, neurovascular coupling results in a hemodynamic response that reflects the location and magnitude of neural activity, which allows us to use oxy- (ΔHbO) and deoxy-Hb (ΔHbR) as markers for brain activity (Kim et al., 2017).

Along with its tolerance to motion, assuming movement of the head is limited (Izzetoglu et al., 2005), strong correlation with fMRI in measurements of hemodynamic responses (Kleinschmidt et al., 1996; Ferrari and Quaresima, 2012), better spatial resolution than MEG and EEG as well as better temporal resolution than fMRI and PET (Bunce et al., 2006; Irani et al., 2007), fNIRS is ideal to study cognitive activity during behavioral tasks in ecologically relevant conditions (Kim et al., 2017), including in children (Caçola et al., 2018). Therefore, it is appropriate for investigating different conditions of the ToH including both three-dimensional (3D) and two-dimensional (2D) modalities.

Our specific aim was to determine if PFC hemodynamics and behavioral performance differ as a function of ToH condition (3D vs. 2D) in neurotypical young adults. We hypothesized that there would be greater changes in ΔHbO during the 3D condition as compared to the 2D condition of the ToH task (H1). Additionally, we hypothesized that each condition would show regional areas with greater changes in oxygenation than other regions within the same condition (H2).

## Materials and Methods

### Participants

Twenty-seven neurotypical adults aged between 18 and 35 were initially recruited for this study from the University of Delaware and Newark communities. Participants were included if they were healthy, right handed, and without known neurological deficits. Three participants were not included due to being left-handed. Exclusion criteria included any head injury within the 6 months prior to testing, an open wound on the forehead, or a seizure disorder. After data collection, an additional three participants were excluded due to incomplete data sets and one participant was excluded due to being an outlier, i.e., hemodynamic data was over two standard deviations from the mean, leaving a total of *n* = 20 neurotypical adults (10F/10M) with a mean age of 24.6 (SD ± 2.8) years old. To ensure that participants were right hand dominant, they completed the Edinburgh Handedness Inventory to assess hand preference for various tasks, such as writing (Oldfield, 1971). Participants were all right-handed and had a mean Laterality Quotient (L.Q.) of 79.2 (SD ± 19.6) with an average Decile of 5.4 (SD ± 3.3; Oldfield, 1971). Sixteen participants had no previous experience solving the ToH puzzle, i.e were task naïve, and the remaining four were self-described beginners, meaning they have had minimal experience solving the ToH puzzle. Participants averaged 172 (±9.61) cm in height and 73.91 (±14.77) kg in body mass. The Institutional Review Board at the University of Delaware approved the protocol for this study and participants provided written informed consent after being educated on the study and its procedures.

### Experimental Design

#### Conditions

**Figure 1:**
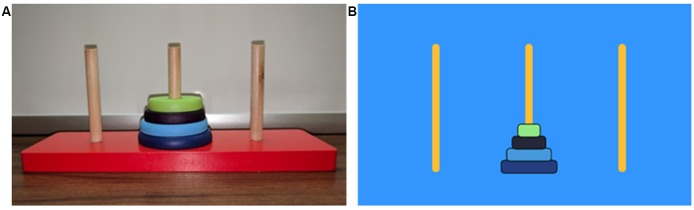
Different Tower of Hanoi (ToH) conditions. **(A)** Three-dimensional (3D) condition performed by using one hand to move disks and the other hand to hold the frame. **(B)** Two-dimensional (2D) condition performed on a PC computer using one hand to manipulate a mouse.

The present study used two conditions: a 3D and a 2D modality of the ToH task. The first condition involved the use of a 3D wooden model, with three peg-holes and four graduated disks, which was physically manipulated during puzzle solving (Figure 1A). The second condition involved the use of a 2D computer model with custom-made opensource software (Salesforce Company, 2018), also consisting of three peg-holes and four graduated disks however manipulated through a computer mouse by clicking and dragging (Figure 1B). Block order (B1/B2) was randomized and counterbalanced using an online randomized integer sequence calculator (Haahr, 1998) resulting in eight participants initially performing the 3D condition (3D/B1) and 12 participants initially performing the 2D condition (2D/B1).

### Protocol

Participants became familiar with the ToH puzzle by performing a practice attempt for each condition using three disks; there were no time constraints for this practice. Following practice, participants had a 1-min rest period, in which they comfortably sat on a chair and focused their attention on a green cross on a screen in front of them, with their eyes open and sitting as still as possible. Following the initial rest period, participants attempted to solve ToH puzzles in two blocks (one per condition; block 1 = B1 and block 2 = B2), each consisting of three 1-min epochs with a 20-s rest in between to allow hemodynamic flow to return to baseline (Kuhtz-Buschbeck et al., 2003; Abibullaev et al., 2014; Yin et al., 2015; Huhn et al., 2019; Table 1). Using Welsh and Huizinga’s (2001) ToH-Revised list containing 22 items, 10 different puzzle configurations were created. Each puzzle had a unique combination of start and end disk positions, which in turn were ordered into two different sequences to avoid an order effect. Puzzles varied in difficulty, with the minimum number of moves required to solve a puzzle ranging from 8 to 15 (Supplementary Figures S1–S3 show the two sequences of 10 puzzles used in this study with associated difficulty). Furthermore, puzzle sequence was randomized and counterbalanced. Each participant was presented with the same puzzle sequence for both 3D and 2D conditions, i.e., they were presented with different puzzles in each sequence but the sequence was identical for both blocks, allowing for a second attempt at a given puzzle if the previous puzzle was solved. After each block, participants had a 1-min rest period.

**Table 1:** Protocol timeline for 3D/B1 participants.

Practice		Rest	Block 1					Rest	Block 2					Rest
3D	2D	R	3D-1	R	3D-2	R	3D-3	R	2D-1	R	2D-2	R	2D-3	R
-	-	60 s	60 s	20 s	60 s	20 s	60 s	60 s	60 s	20 s	60 s	20 s	60 s	60 s

*Participants were introduced to the puzzle with a practice attempt for each condition, followed by a 1-min rest period prior to completing two blocks of problem-solving. 3D, three-dimensional ToH condition; 2D, two-dimensional ToH condition; R, rest*.

### Experimental Device

A 16-channel continuous-wave functional near-infrared (fNIRS) device (Ayaz et al., 2012, 2013) was utilized to collect data from the PFC of all participants (fNIR Devices LLC, Potomac, MD, USA). The sensor included 10 photo detectors and four light emitters (16 optodes total), each releasing light at 730–850 nm wavelengths and separated by 2.5 cm, which allowed for a penetration depth of about 1.2 cm (Ayaz et al., 2011).

**Figure 2:**
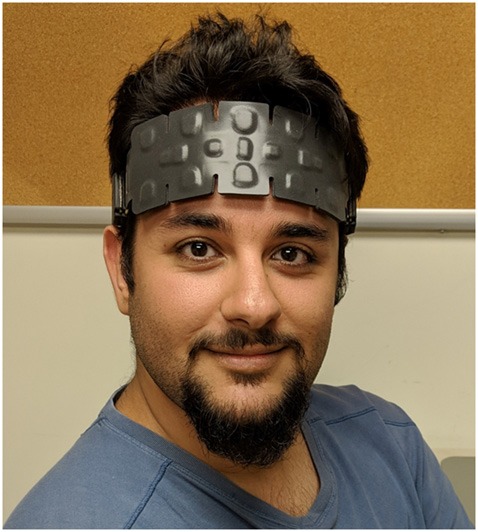
fNIR^®^ sensor pad used for data collection. Written informed consent was obtained from the individual to publish this image.

**Figure 3:**
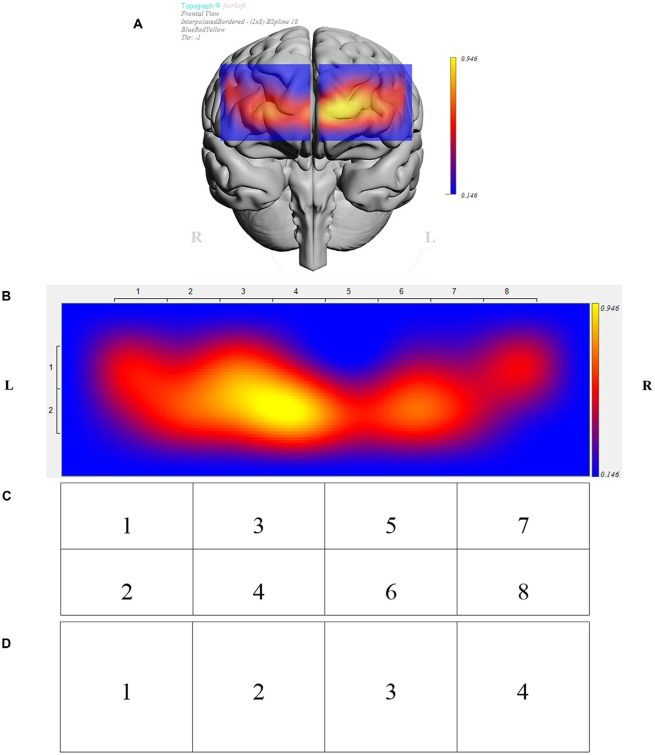
**(A)** Brain template with data from sample participant depicting prefrontal regions under investigation. **(B)** Brain activity presented in 16 measurements locations (optodes) for the same participant. Data was further analyzed in clusters of eight regions **(C)**, and four regions **(D)**. **(B–D)** Depict opposite laterality from panel **(A)**, i.e., the right side of the figure corresponds to the right hemisphere and vice versa.

The fNIRS sensor band was placed on top of the participants’ forehead such that the center of the sensor’s horizontal axis was aligned to the center of the participants’ head (symmetry axis of the head). The sensor’s vertical axis was positioned in the Fp1 and Fp2 locations in accordance to the international 10-20 system of cerebral electrode placement (Homan et al., 1987; Ayaz et al., 2006). Figure 2 depicts the fNIRS sensor band used in this study (fNIR Devices LLC, Potomac, MD, USA) and Figure 3 shows an fMRI-based topographic map of the brain (Ayaz et al., 2018).

### Data Acquisition and Analysis

fNIRS data were sampled at 2 Hz and acquired using Cognitive Optical Brain Imaging (COBI) studio software (Ayaz et al., 2011). Data were processed using fNIRSoft Software (Version 4.9, Ayaz et al., 2018) and represents the mean activation during all three 1-min blocks for each condition. The device was initiated and the first 10 s were utilized as baseline prior to task initiation. During this time, the participant remained still and focused on a green cross located in a computer screen across from the participant. Before placing the sensor band, an alcohol swab was used to clean the forehead of the participants and the light in the testing room was subsequently reduced. Researchers were careful to exclude any hair between the sensors and the participants’ forehead to obtain optimal signal acquisition.

Raw light intensities were visually inspected and individual optodes rejected when data did not reflect hemodynamic activity due to lack of proper contact between the sensors and the forehead or placement on top of hair. Researchers also visually inspected the data and manually removed optodes in accordance to Ayaz et al. (2011). Then, a finite impulse response (FIR) filter (20th order, Hamming window) was used to low-pass filter the raw light intensity data at 0.1 Hz to remove input from physiological signals, such as respiration and heartbeat. Data were subsequently converted to changes in concentration using the modified Beer-Lambert law (Cope and Delpy, 1988) and depicted into four outcome measures: change in oxygenated hemoglobin (ΔHbO), change in deoxygenated hemoglobin (ΔHbR), total change in hemoglobin (ΔHbT) and total change in oxygenation (ΔOxy). We restricted our analysis to ΔHbO because our preliminary analysis showed high correlation among ΔHbO, ΔHbT, and ΔOxy, in addition to ΔHbO having stronger and more wide-spread signals than those from ΔHbR and ΔHbT (Zhang et al., 2011), including in the ToH task (Liang et al., 2016a,b). Furthermore, it has been shown that ΔHbO has a strong correlation to BOLD signal whereas ΔHbR has a weak correlation (Strangman et al., 2002), in addition to better signal-to-noise ratio (SNR) than ΔHbR (Hoshi, 2007; Zhang et al., 2010). Finally, data depicting changes in concentration were processed using the detrending filter, which removes a drift in the data using linear parameters that convert the slope of the baseline to zero.

Hemodynamic data for 20 participants were included in the analyses and reflected a mean activation of the three 1-min epochs for each condition. Furthermore, performance data for 17 participants were included in the analyses, defined as the number of puzzles a participant attempted to solve within each block out of 10 potential puzzles. Due to data collection error, performance data were not obtained for three participants.

### Statistical Analysis

Data were analyzed in JMP^®^ Pro 13.1.0. The effects of 2D vs. 3D conditions, presentation order (leading block), individual participants, and regions were examined using a mixed effects ANOVA with participants nested within blocks. A separate analysis was conducted for each of four representations of the optodes based on the sensor layout (Figures 3B–D). Those four representations depicted data for the complete PFC (overall), a division of the PFC into four regions (left lateral, left medial, right medial, and right lateral), a division of the PFC into eight regions (left lateral dorsal and ventral, left medial dorsal and ventral, right medial dorsal and ventral, and right lateral dorsal and ventral), and a division of the PFC into 16 regions, i.e., data for each of the individual optodes. An exploratory analysis subsequent to ANOVA investigated the significance of conditions by considering paired differences between 2D and 3D for each participant and each optode representation in Tukey mean difference plots. A regression analysis showed the association between performance (puzzles attempted) and ΔHbO.

## Results

### ΔHbO in 2D vs. 3D Conditions

**Figure 4:**
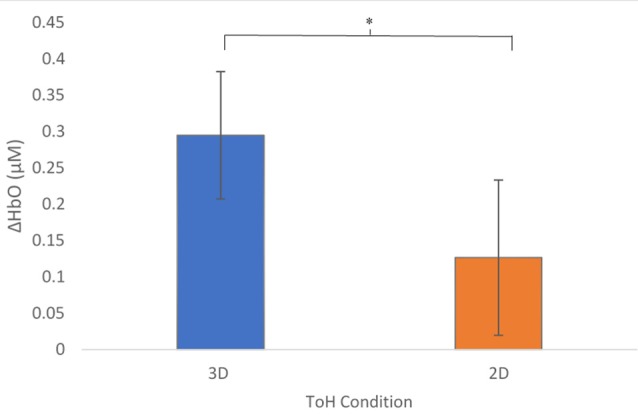
Mean Changes in oxygenated hemoglobin, ΔHbO (μM) as a function of condition. The 3D condition showed significantly higher activity, *p* = 0.0207. Error bars show standard error values. *denotes statistical significance.

**Figure 5:**
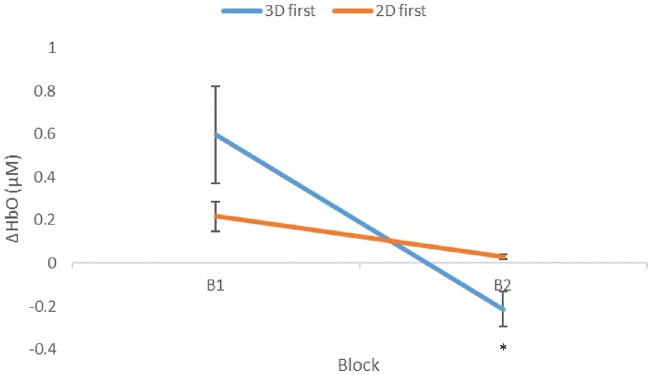
Mean ΔHbO (μM) as a function of presentation order. Condition changes from first (B1) to second (B2) block. Significantly lower mean ΔHbO was seen in B2 with respect to B1 for participants that initially performed the 3D condition, *p* = 0.0015, shown as 3D first (3D/B1 group). Error bars show standard error values. *denotes statistical significance.

A mixed-effects ANOVA based on a mean ΔHbO response over all optodes was performed with conditions, presentation order, participants within blocks, and region as factors. As predicted, overall ΔHbO was significantly higher for the 3D condition compared to the 2D (Figure 4, *p* = 0.0211). In addition, a significant interaction existed between the order of presentation and condition (Figure 5, *p* = 0.0015), with those participants who started with 3D in block 1 (3D/B1) showing a much larger change in ΔHbO than those who started with 2D (2D/B1). Additionally, participant responses to experimental conditions were not universal: there were significant interactions between condition and participant (*p* = 0.0013), region and participant (*p* = 0.0124), and there was a three-way interaction of participant × region × block order (*p* < 0.0001).

### ΔHbO and Regional Analysis

With a similar mixed-effect ANOVA analysis using individual optode recordings for ΔHbO, no significant differences were seen in regional activity using the 16, 8, or 4 optode model with this particular group (*p* > 0.05). In other words, specific sets of optodes did not show higher ΔHbO activity within a condition.

### ΔHbO and Performance on ToH

ΔHbO decreased significantly between the first and second block (*p* = 0.0015); at the same time, the total number of puzzles solved increased significantly for both 3D and 2D conditions (B1 = 3.56, B2 = 4.50, *p* < 0.005). Furthermore, there was a negative correlation between the number of puzzles solved and ΔHbO (*r* = −0.37), suggesting that participants learned the ToH task and could perform it with less cognitive effort in the second block.

**Figure 6:**
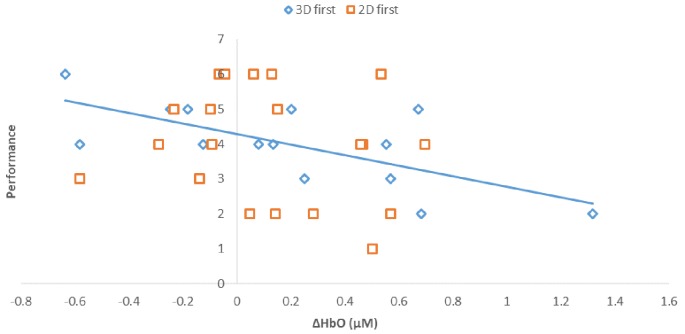
Correlations between performance (# of puzzles attempted to solve) and prefrontal activity (ΔHbO, μM). Regression line shows the strong correlation, *r^2^* = 0.473, *p* < 0.01, found in participants that initially performed the 3D condition, shown as 3D first (3D/B1 group).

**Figure 7:**
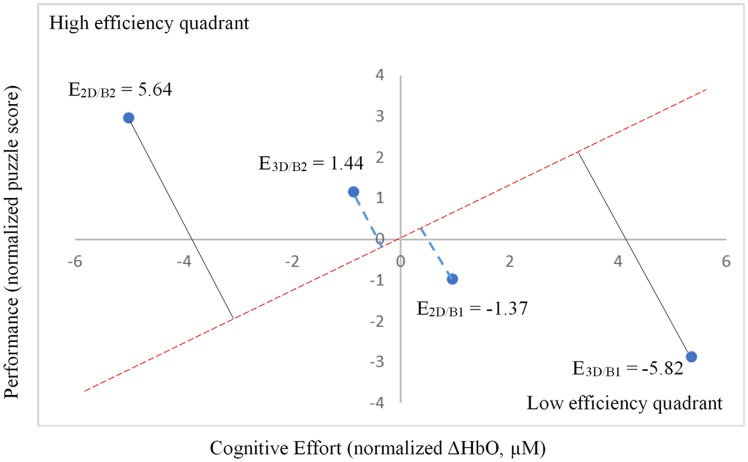
Relative neural efficiency as measured by performance (normalized puzzle score) and cognitive effort (normalized ΔHbO, μM).

Notably, when the 3D condition was performed in block 1 (3D/B1), a significant negative correlation between performance and ΔHbO existed (*r* = −0.69, *r*^2^ = 0.473, *p* < 0.01, Figure 6). In contrast, when participants started with the 2D condition, there was no significant correlation between B1 and B2 (*r* = −0.177, *r*^2^ = 0.0313, *p* > 0.05) when the 2D condition was performed in block 1 (2D/B1; Figure 6). Due to the significant interaction found between conditions (2D vs. 3D) and condition order (leading block) along with the correlation differences, we followed up with an analysis of relative neural efficiency (Paas and Van Merriënboer, 1993; Paas et al., 2003). Both groups improved relative neural efficiency from B1 to B2, but the amount of change differed substantially. The calculated relative neural efficiency for 3D/B1 participants was −5.82 for block 1 and 5.64 for block 2. In contrast, the 2D/B1 participants had a relative neural efficiency of −1.37 for block 1 and 1.44 for block 2 (Figure 7).

## Discussion

### Changes in Oxyhemoglobin as a Function of Puzzle Type

As we hypothesized, ΔHbO was significantly higher for the 3D condition compared to the 2D condition overall (Figure 4). This suggests that the 3D condition placed higher cognitive demands on the participants in order to solve the puzzle, which may be due to greater demands on perceptual decision-making processes (object identification, selection, and localization) due to the integration of increased spatial and tactile sensory processing (Wong et al., 2015). Additionally, higher demands on motor planning, i.e., increased demands on abstract kinematics, action selection, and movement (Wong et al., 2015), would be imposed by the 3D condition with respect to the 2D condition.

Furthermore, participant responses to experimental conditions varied on the basis of condition, presentation order, and region, as demonstrated by the significant interactions (condition and participant, region and participant, participant × region × presentation order). These results provide further support for individual differences in EF (Ackerman, 1987, 1988; Miyake et al., 2000; Osaka et al., 2004; Miyake and Friedman, 2012; Smith et al., 2019) since participants had different levels and patterns of neural activity for each condition between each other (interpersonal differences) and amongst themselves (intrapersonal differences). Given that performance in the ToH task has been associated to inductive reasoning as well as other aspects of fluid intelligence (Welsh et al., 1999; Devine et al., 2001; Lock et al., 2002), the study done by Dunst et al. (2014) is of relevance. Brain activity between “brighter” and “less bright” participants was compared as they worked on a number-series task taken from the numerical-inductive reasoning (Arendasy and Sommer, 2012) of the intelligence-structure-battery (INSBAT, Arendasy, 2008). They found significant differences in brain activity within eight regions (particularly left inferior frontal, left middle frontal, and right middle frontal), when the task difficulty was not standardized, i.e., adjusted for each participant according to their ability based on intelligence scores. Our study did not standardize the ToH task difficulty based on participants’ ability, thus individual differences can be partially explained by ability in terms of fluid intelligence.

Moreover, it has been suggested by Simon (1975) that the restrictions of the ToH task lead to the creation of various problem-solving strategies with varying effectiveness and that can account for individual differences, particularly for performance. Furthermore, Simon (1975) proposes the recursive guiding solution method as the optimal problem-solving strategy since it decreases the demands placed on working memory, planning, and inhibition (all executive processes) by the inherent complexity of the ToH puzzle. The recursive guiding solution begins with moving the largest disk to the end-goal position, followed by moving the smaller disk out of the way and in the open peg (subpyramid stack of smaller disks), and then restarting the process by moving the largest disk in the subpyramid to the end-goal position and repeating all steps until reaching the solution (Simon, 1975).

### Prefrontal Cortex: Overall vs. Regional Differences

Contrary to our hypothesis and despite previous studies demonstrating that hemodynamic responses can be used to reliably quantify cognitive state and load levels in the bilateral PFC (Basso Moro et al., 2013; Fishburn et al., 2014; Unni et al., 2015; Bonetti et al., 2019), the present study did not find significant differences among optodes when parsed into different regions. Although the lack of regional differences is important, it is worthwhile to note that fNIRS’ spatial resolution is limited by the optical source-detector distance, which is normally 2–3 cm, despite the technology having high temporal resolution (Tak et al., 2016). This relatively low spatial resolution, compared to fMRI, may cause difficulties in localizing different regions of the brain. Additionally, this study analyzed regional differences based on the layout of the device (4, 8, and 16 regions), which may not have been appropriate to parse out the functional activity of the PFC.

Moreover, the present study did not stratify participants based on individual differences shown to reflect distinct brain activity, such as fluid intelligence and spatial ability (Lamm et al., 2001; Neubauer and Fink, 2009; Dunst et al., 2014). The presence of individual differences, as described above, might have precluded our ability to detect regional differences. Lastly, previous research has shown that restricting the processing time during a cognitive task can affect the magnitude and patterns of neural activity (Porebski, 1954; McCarthy and Wood, 1985; Van Breukelen and Roskam, 1991; Haig et al., 1997; Gulliksen, 2013), and even increase the demand for neural resources (Lamm et al., 2001). It could be that restricting the time to complete puzzles to 1-min epochs affected our study in unexpected ways. Other analysis techniques that take into consideration temporal aspects of each epoch, such as a time-series analysis, might lead to identification of differences.

### Associations Between Brain and Behavior

There may have been a learning effect across the blocks, as ΔHbO decreased significantly between B1 and B2 while the total number of puzzles solved increased significantly, regardless of condition order. Since prior knowledge reduces the cognitive load inherent to task complexity, also known as the intrinsic load (Kalyuga et al., 2004; Sweller, 2008), having solved the same puzzles in B1 as in B2 likely reduced this intrinsic load placed by the puzzle in B2. This shift in cognitive load from B1 to B2 led to an approximation of the “ideal” load for the task, also known as germane cognitive load, thereby reducing the amount of neural resources needed to solve the puzzle (Sweller, 2008; Taylor, 2013). This is further supported by the negative correlation seen between the number of puzzles solved and ΔHbO, suggesting that participants may have progressively learned to solve the ToH task and could perform it with less cognitive effort in B2 due to a more efficient use of neural resources.

Our results support evidence from previous studies in neural efficiency, listed in the review by Neubauer and Fink (2009), where a negative relationship between brain activation and task performance has been consistently seen on tasks of low to medium difficulty or complexity, indicating a potential association between performance and cognitive load. Likewise, our results further support previous evidence where practice has led to a significant negative correlation between performance and neural activation, indicating a more discriminating activity of neural circuitry in B2 due to a sharpened cognitive strategy gained during B1 (Haier et al., 1988, 1992).

As previously mentioned, presentation order (leading block) and condition interacted significantly (Figure 5), showing a significant negative correlation between performance and ΔHbO (Figure 6) for 3D lead participants (3D/B1 group) as opposed to 2D lead participants (2D/B1 group). A relative neural efficiency analysis (Paas and Van Merriënboer, 1993; Paas et al., 2003) showed that while both groups improved relative neural efficiency from B1 to B2, the magnitude of change was considerably different, with those starting with the 3D condition showing increases in neural efficiency that were five times greater than those starting with 2D. This suggests that learning the ToH using the 3D puzzle, while more difficult, leads to higher cognitive efficiency in subsequent trials. Because of the task design, it is unclear if this improvement in neural efficiency would occur if the participants continued to perform the task manually.

As previously mentioned, 2D/B1 participants did not show a significant correlation between B1 and B2. Since a significant negative correlation between performance and neural activity indicates a shift towards higher neural efficiency, it can be concluded that participants in the 2D/B1 group did not learn the task, at least not as well as 3D/B1 participants. While this is an immediate effect given our study’s paradigm, our results are consistent with the study by Kantak et al. (2017) where they found that practicing a complex skill improves the long-term performance of the unpracticed simpler goal-directed task. The complex task presents the participant with higher motor and/or cognitive load, which in turn increases neuroplastic changes across the cognitive-motor network, essential for learning and its transfer (Nudo et al., 1996; Plautz et al., 2000; Meehan et al., 2011; Lefebvre et al., 2015; Wadden et al., 2015).

The improvement in neural efficiency in the 3D/B1 group may be a result of a contextual interference effect, where initial high contextual interference during skill acquisition leads to an initial decrement in performance followed by better performance once the skill is learned, as compared to a low interference task (Lee et al., 1992). In this study’s paradigm, the change in performance from B1 to B2 was greater when the 3D ToH condition was performed, along with the highest overall ΔHbO and subsequent lowest overall ΔHbO in the 2D ToH condition. Thus, having a more complex modality of a task, i.e., a 3D puzzle, would place higher demands on sensory information processing in addition to motor planning demands, which can be seen as interfering with the task itself and therefore increasing cognitive load. This result is analogous to that of Welsh and Huizinga (2005) where two sets of participants were administered either a blocked schedule of increasingly difficult ToH puzzles, or a random schedule with randomized order of puzzle difficulty. They found that as expected, the blocked group had a decrease in performance, whereas the random group increased their accuracy thereby evidencing a learning effect (Welsh and Huizinga, 2005).

### Limitations and Future Directions

One limitation to this study is that participants were not stratified by individual differences, particularly differences that have been shown to reflect distinct brain activity such as fluid intelligence and spatial ability (Lamm et al., 2001; Neubauer and Fink, 2009; Dunst et al., 2014). Future work can address this limitation by creating distinct groups based on relevant individual differences amongst the participants. Additionally, future work can utilize other analysis techniques that take into consideration temporal aspects of each epoch, in order to further assess for regional differences, and expand analysis by using a system that allows to visualize more regions of the brain.

## Conclusion

To our knowledge, this is the first study that investigated the neural activity of the PFC while controlling the cognitive component and manipulating movement, using a 3D and 2D modality of the ToH puzzle. To answer the question posed by our title, it appears that, in fact, movement does matter. Our results showed higher oxyhemoglobin concentration for the 3D condition compared to the 2D condition, suggesting a higher complexity for the 3D condition due to increased demands on sensory processing and integration as well as motor planning. Practice and the order of presentation contributed to a learning effect, where oxyhemoglobin decreased and performance increased from B1 to B2 overall, however, the relationship was significant for the 3D/B1 group unlike the 2D/B1 group. This suggests that not only practice is important for performance, but also the complexity of the task first performed which in turn leads to a refined cognitive strategy as shown by the considerable decrease in oxyhemoglobin concentration from B1 to B2 in the 3D/B1 group. Overall, our results further support previous evidence by showing that neural efficiency eases demands on EF, although it is contingent on task constraints. Our results also provide further evidence in the importance of practicing a more complex version of a task prior to executing a simpler version for learning and performance. We plan to expand our exploration of PFC activity between the two- and three-dimensional ToH conditions to children to narrow the potential points of impairment on the perception-cognition-action continuum in certain developmental disabilities.

## Ethics Statement

All subjects gave written informed consent in accordance with the Declaration of Helsinki prior to participation. The protocol was approved by the Institutional Review Board at the University of Delaware.

## Author Contributions

NG and KM designed the experiment. KM and EB collected the data. KM and BB analyzed the data and prepared all figures. KM, EB and NG drafted the manuscript. NG and BB provided critical revisions. All authors approved the manuscript to be published.

## Conflict of Interest Statement

The authors declare that the research was conducted in the absence of any commercial or financial relationships that could be construed as a potential conflict of interest.
