# Supplementary material for: Does Movement Matter? Prefrontal Cortex Activity During 2D vs. 3D Performance of the Tower of Hanoi Puzzle
Source: Front Hum Neurosci. 2019 May 10;13:156. doi: 10.3389/fnhum.2019.00156 (PMC6539212; doi:10.3389/fnhum.2019.00156)
Supplement: Supplementary file 1 [file Data_Sheet_1.PDF]

Supplementary Material

1 Supplementary Figures

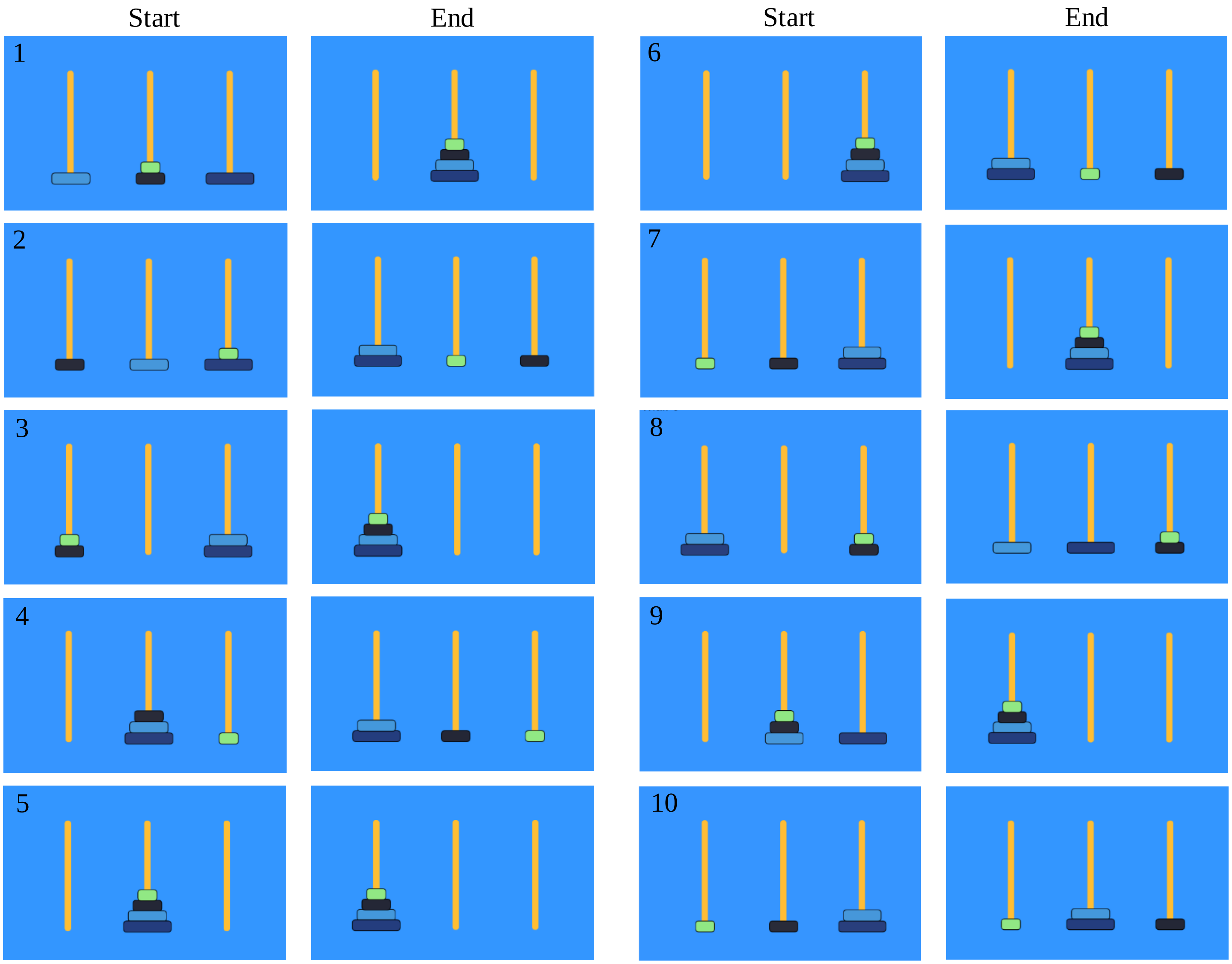

Supplementary Figure 1. Sequence 1 containing the ten ToH puzzles utilized in this study. ToH puzzle sequences were created based on Welsh and Huizinga's (2001) Tower of Hanoi-Revised list containing 22 items. Start = starting position, End = end goal position.

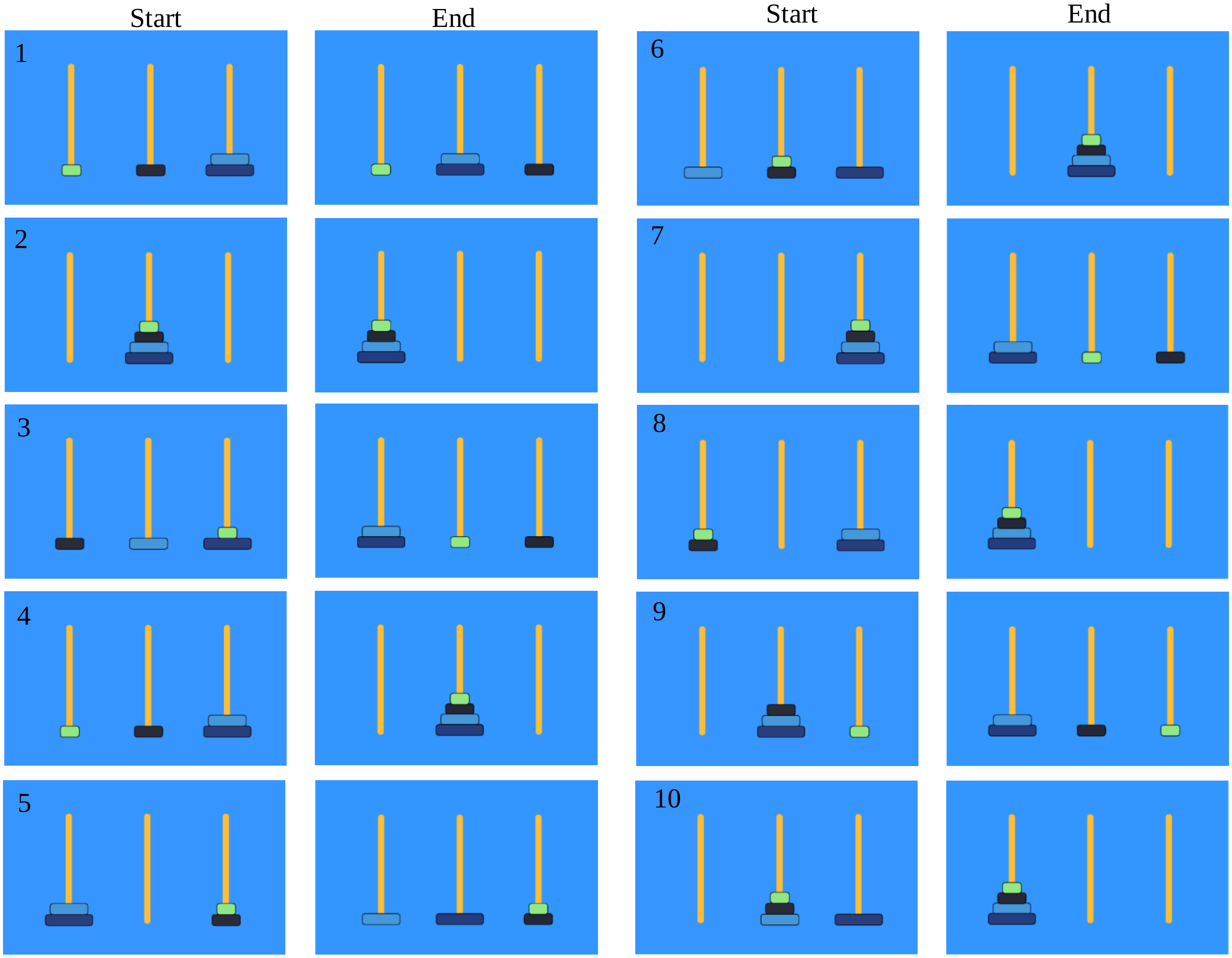

Supplementary Figure 2. Sequence 2 containing the ten ToH puzzles utilized in this study. Start = starting position, End = end goal position.

Sequence 1	
Puzzle number	Minimum # moves to solution
1	11
2	8
3	12
4	12
5	15
6	13
7	13
8	15
9	8
10	11

Sequence 2	
Puzzle number	Minimum # moves to solution
1	11
2	15
3	8
4	13
5	15
6	11
7	13
8	12
9	12
10	8

Supplementary Figure 3. Difficulty for each ToH puzzle represented as the minimum number of moves necessary to reach the solution (end goal position). Both sequences contain the same puzzles but presented in a different order.
